# Passive Surveillance of Malaria in Pregnant Women, Non-pregnant Women and Children Under 5 Years of Age in Bannu District, Khyber Pakhtunkhwa Pakistan

**DOI:** 10.3389/fmed.2021.751456

**Published:** 2021-11-18

**Authors:** Humera Qureshi, Muhammad Imran Khan, Akhlaq Ahmad, Usman Ayub Awan, Aamer Ali Khattak, Ayesha Ali Khan, Yaqiong Sun

**Affiliations:** ^1^Department of Mathematics and Statistics, The University of Haripur, Haripur, Pakistan; ^2^Gungdong Provincial Key Laboratory of Allergy and Clinical Immunology, The State Key Laboratory of Respiratory Disease, The Second Affiliated Hospital, Guangzhou Medical University, Guangzhou, China; ^3^Department of Medical Laboratory Technology, The University of Haripur, Haripur, Pakistan; ^4^Department of Biochemistry and Molecular Biology, Quaid-i-Azam University, Islamabad, Pakistan; ^5^Department of Imaging, Southern University of Science and Technology Hospital, Shenzhen, China

**Keywords:** malaria, infection, pregnant women, Bannu, Pakistan

## Abstract

**Background:** Malaria among pregnant women is one of the major causes of maternal and infant mortality and morbidity, especially in high-risk areas. Therefore, our study identified the burden of malaria for pregnant women, non-pregnant women, and children under 5 years of age, and malaria service health facilities in Bannu district, Khyber Pakhtunkhwa, Pakistan.

**Methods:** A cross-sectional study was conducted. In this survey, 15,650 individuals were surveyed, and 1,283 were malaria-positive detected. The data were collected from 80 different healthcare centers. SPSS version 23 was used for data analysis. ArcGIS version 10.8 was used for study area mapping.

**Results:** Malaria was detected in 23.3% of children under five, 4.4% of pregnant women, and 72.3% of non-pregnant women, respectively. Moreover, *P. falciparum, P. vivax*, and mixed infection had a prevalence of 2.1, 96.8, and 1.1%. The most often used and effective medications to treat malaria were chloroquine (29.7%) and primaquine (69.4%).

**Conclusion:** This study's findings depict that malaria's prevalence in the non-pregnant women's group was high. Additionally, *P. vivax* infection was found to be more prevalent than other types of malaria infection. Due to the scarcity of healthcare facilities in this endemic region, special attention should be directed to strengthening the malaria surveillance and eradication programs.

## Introduction

Malaria among pregnant women is one of the main causes of maternal and infant mortality and morbidity, especially in high-risk areas (1). About 125 million pregnant women are likely to become infected with malaria during pregnancy, especially in Africa, where *Plasmodium falciparum (P. falciparum)* is the most important infection. Similarly, *Plasmodium vivax (P. vivax)* is a major threat to pregnant women: about 90 million are susceptible every year in Asia. With 300 to 600 million cases occurring every year and almost 2.2 billion people at high risk, malaria is a global concern (2).

The complications of malaria during pregnancy about 10,000 pregnant women and 200,000 newborns deaths occur every year (3). About 50 million women live in areas with high malaria incidence each year (4). During gestation, malaria can cause adverse outcomes in the newborn baby, including fetal growth restriction/small for gestational age infant, miscarriage, low birth weight, pre-term birth, perinatal death, and congenital and postpartum infection (5). Pregnant women are more vulnerable than non-pregnant women to growing problems, such as pulmonary edema, acute respiratory distress syndrome, and hypoglycemia (6–8). Around 30 million pregnant African women are in danger of malaria infection, whereas 56 million suffer from anemia (9, 10). *P. falciparum* is the most common cause of severe clinical manifestations among the other malaria species, leading to maternal anemia, low birth weight, and pre-term birth. *P. vivax* infection, on the other hand, is essential and can result in the same complications as *P. falciparum* infection, but it is less prevalent (5). The most significant clinical manifestations are not specific and variable. The primary symptom of malaria is fever, which is frequently accompanied by chills and excessive sweating, myalgia, nausea, headache, fatigue, vomiting, abdominal pain, and diarrhoea (4).

Malaria transmission is seasonal, and specific geographical areas of Pakistan, primarily Khyber Pakhtunkhwa, Sindh, and Balochistan, are prone to outbreaks. However, in Khyber Pakhtunkhwa province, three malaria-endemic regions are more vulnerable: Bannu, Dera Ismail Khan, and Lakki Marwat (11). The *P. vivax* high transmission occurs between June and September and between April and June (12). Similarly, the predominant period of transmission of *P. falciparum* in Pakistan is August to December (11, 13). With 88 and 12%, *P. falciparum* and *P. vivax* are Pakistan's most prevalent malarial species. The most significant malaria prevalence observed in the FATA area, and Pak-Afghan and the Iranian border was 37% (14). The National Malaria Control Program (MCP) ranked FATA third in malaria burden-sharing after Sindh and Khyber Pakhtunkhwa (KP) (12). Nonetheless, FATA had the highest yearly malaria prevalence and tested positive rate of any province or territory in Pakistan (15).

A majority of symptomatic patients in malaria-endemic areas where diagnostic tests facilities are unavailable are diagnosed using empirical diagnosis, although it leads to overtreatment due to errors in diagnosis (16). Nonetheless, a tentative diagnosis is a most prevalent and least expensive technique for self-treatment in rural areas in Pakistan (17). Amongst two worldwide prominent *Plasmodium* species, *P. vivax* is considered most frequent in Pakistan, accounting for 80% of all cases of malaria, whereas P. falciparum is responsible for infecting about 20% of cases worldwide (18, 19). Malaria detection and monitoring are challenging without a more sensitive, specific and accurate diagnostic approach in areas where even more than one *Plasmodium* species is prevalent (17). Plasmodium detection and speciation methods include microscopy (light and fluorescence), immunochromatography, nucleic acid amplification, and flow cytometry (20). However, a rapid diagnostic test (RDT) was developed to overcome the limitations of microscopy and has been shown to be a fast, efficient, and cost-effective malaria screening procedure (18, 21). According to World Health Organization (WHO) malaria recommendations, all suspected cases should undergo laboratory testing by using microscopy and RDT before receiving therapeutic treatment (21).

This study was conducted in the Bannu district of Northern Pakistan, which has a higher rate of malaria transmission than the national norm. Bannu is the major contributor of malaria cases in southern districts after Dera Ismail Khan and Lakki Marwat (11), with approximately one million people presently living in areas with a high risk of malaria transmission. There is paucity of available studies on the prevalence of malaria in pregnant women on nationwide scale. Therefore, current study aim is to determine the malaria prevalence in pregnant women, non-pregnant women, children under the age of five, and healthcare facilities in Bannu district, Khyber Pakhtunkhwa, Pakistan.

## Methods

### Study Design, Area and Period

**Figure 1:**
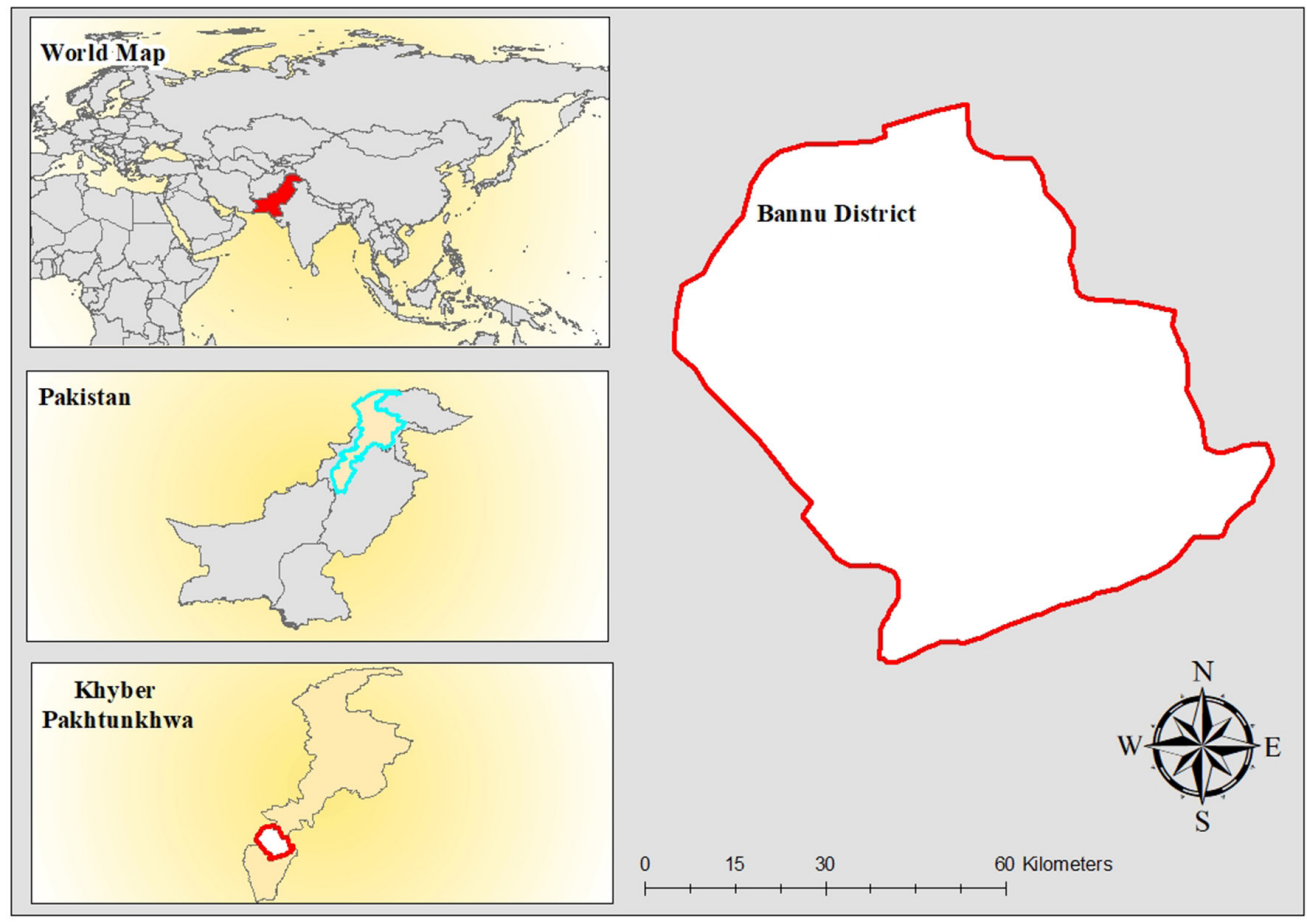
Study area map.

This cross-sectional study was conducted in the Bannu endemic district Figure 1, and the estimated total population of the entire study area is 1,218,416. The current study report from August to October 2018, which was the highest season for malaria transmission. It exhibits all of the characteristics of a plain because of sand dunes, scorching temperatures, and dry weather. Summers are sweltering, while winters are just marginally cooler. Early April is usually the start of summer, and it lasts until the end of the year. The cold snap begins in early November, and the winter season lasts until the beginning of March (22).

Before the conduction of this study, malaria's prevalence has peaked for some reasons, mainly socio-economic, unstable political environment, and internally displaced persons (IDPs) in the neighboring areas from North Waziristan to Khyber Pakhtunkhwa Province (14). A total, 950,000 people faced displacement (73% of women and children) and were resettled in local communities and refugee camps (18).

**Figure 2:**
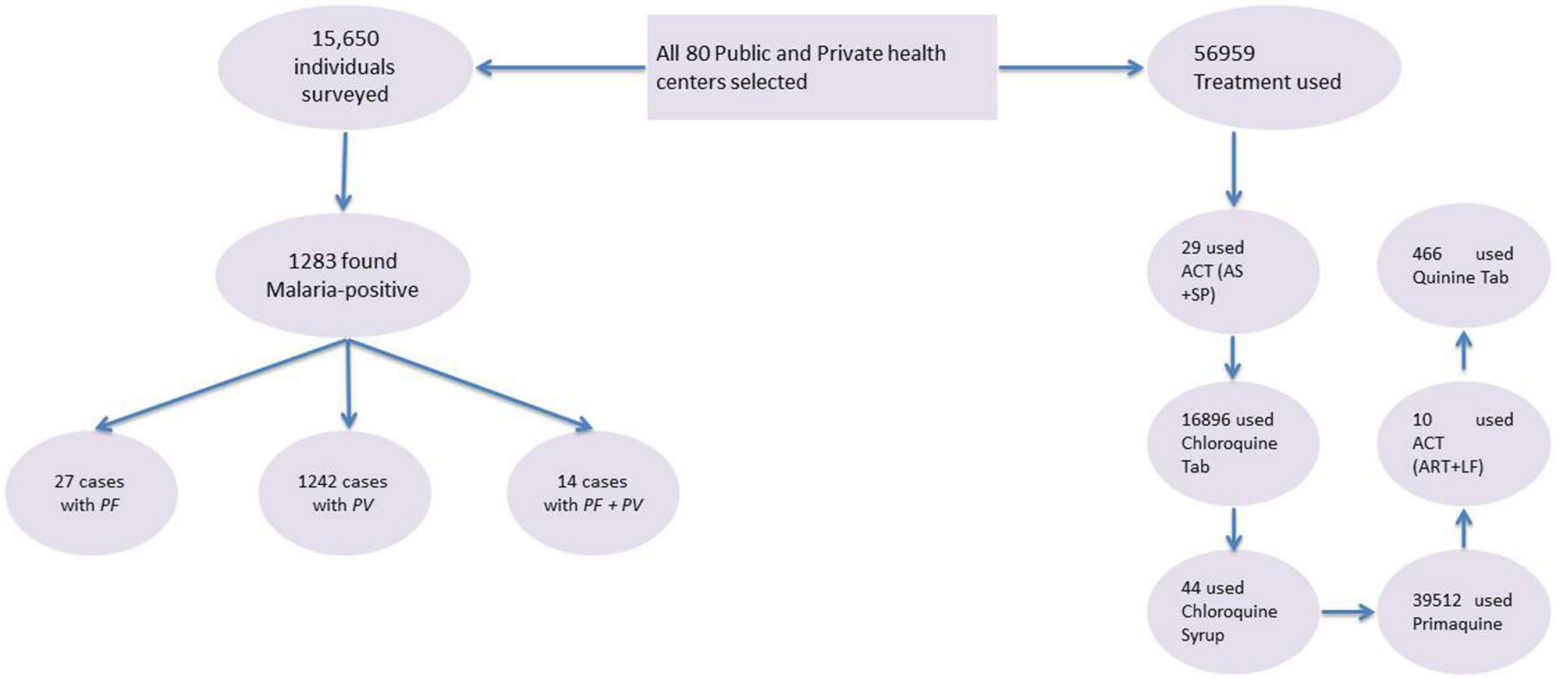
Flow chart of malaria diagnosis in high-risk groups and treatment at health centers. *PF, P. falciparum; PV, P. vivax; ACT (AS*+*SP), artemisinin combination therapy (artesunate* + *sulfadoxine – pyrimethamine); ACT (ART* + *LF), artemisinin combination therapy (artemether* + *lumefantrine)*.

Eighty healthcare centers were equipped with microscopes, rapid diagnostic testing (RDT), and both were surveyed in the Bannu district. In this study, 15,650 people were interviewed, and 1,283 people were found to have malaria, according to the results Figure 2. The strata consisted of urban and rural areas. The statistics on the application were subdivided by gender, age, and kind of malaria parasite infection. ArcGIS version 10.8 was used to conduct an area mapping analysis.

### Cluster Sampling

The cluster sampling technique was carried out where the whole population was divided into clusters, and a random sample of these clusters was selected. All of the observations in the selected cluster are included in the final selection process. It can be understood as simple random sampling as described, except that the primary sampling unit is a cluster or group of people instead of the individual. Cluster sampling is typically used when the researcher cannot get a complete list of the population members. It was also used when a random sample would produce a list of subjects so widely scattered that surveying them would prove far too expensive.

### Data Collection

This study's main data collection technique was a questionnaire that includes two types: a malaria infection registration form for high-risk groups (pregnant women, non-pregnant women, and children under 5 years of age) and a malaria facility service form for healthcare centers. Demographic data, including gender, age, residence, healthcare center name, and personal code, have been merged into the database. The malaria facility service form mainly includes malaria diagnosis information and healthcare center treatment. Pregnant women, non-pregnant women, and children under 5 years of age positive cases were included, while >5 years of age male positive infections and negative cases were excluded from this study.

### Quality Assurance

Different quality control activities were applied to ensure the data collected in this study, such as re-examining the slides, training data collectors, supervision by qualified experts, and monitoring integrity and completeness of data.

### Data Analysis

Data analysis was performed with SPSS version 23 (SPSS Inc. Chicago: IL, USA). Descriptive statistics were given as means (± SD) for continuous variables and as frequency and %age for categorical variables. Chi-square (χ^2^) test was used to explore the relationship between categorical variables. The level of significance was considered at the probability level (*P* ≤ 0.05).

### Ethical Considerations

Ethical clearance was obtained from the University of Science and Technology Bannu, Pakistan vide ethical number USTB/Ethics/Comm./2018/05. Permission to conduct the research and relevant information was obtained by the relevant authorities at the Directorate of the Malaria Control Programme, Pakistan.

## Results

This survey includes 15,650 individuals and found 1,283 (8.2%) were malaria-positive, of which children under 5 years of age, pregnant women, and non-pregnant women were 23.3% (21.0–25.6 CI 95%), 4.4% (3.4–5.6 CI 95%), and 72.3% (69.9–74.7 CI 95%). Table 1 summarizes the basic characteristics of the study population. Information about malaria facilities, such as diagnosis, reporting, and treatment, was obtained from 80 healthcare centers established by governments and international non-governmental organizations (NGOs).

**Table 1:** Demographic characteristics of residents surveyed in Bannu districts (*n* = 15,650).

**Characteristics**	**Total surveyed (N)**	**Positive**	**%**	**95 % CI**	
				**Lower**	**Upper**
**Status**					
<5	4,500	299	6.64	2.00	25.60
PW	3,911	56	1.43	0.40	5.60
Women	7,239	928	12.88	5.90	45.70
Total	15,650	1,283	8.20		

^*^ <5, Below five year children; ^*^PW, Pregnant women.

**Figure 3:**
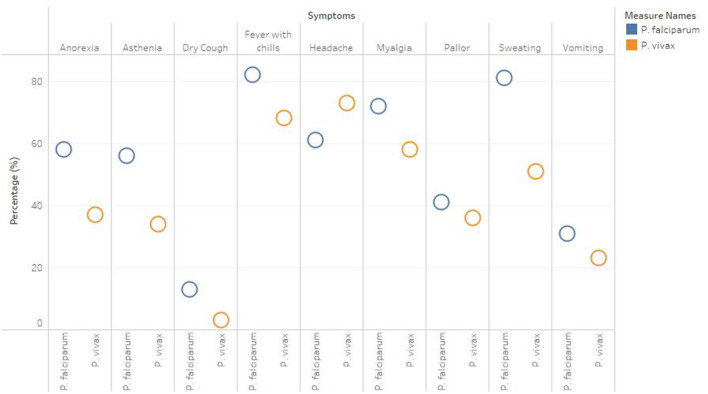
Clinical Manifestation of *P. falciparum* and *P. Vivax*.

**Figure 4:**
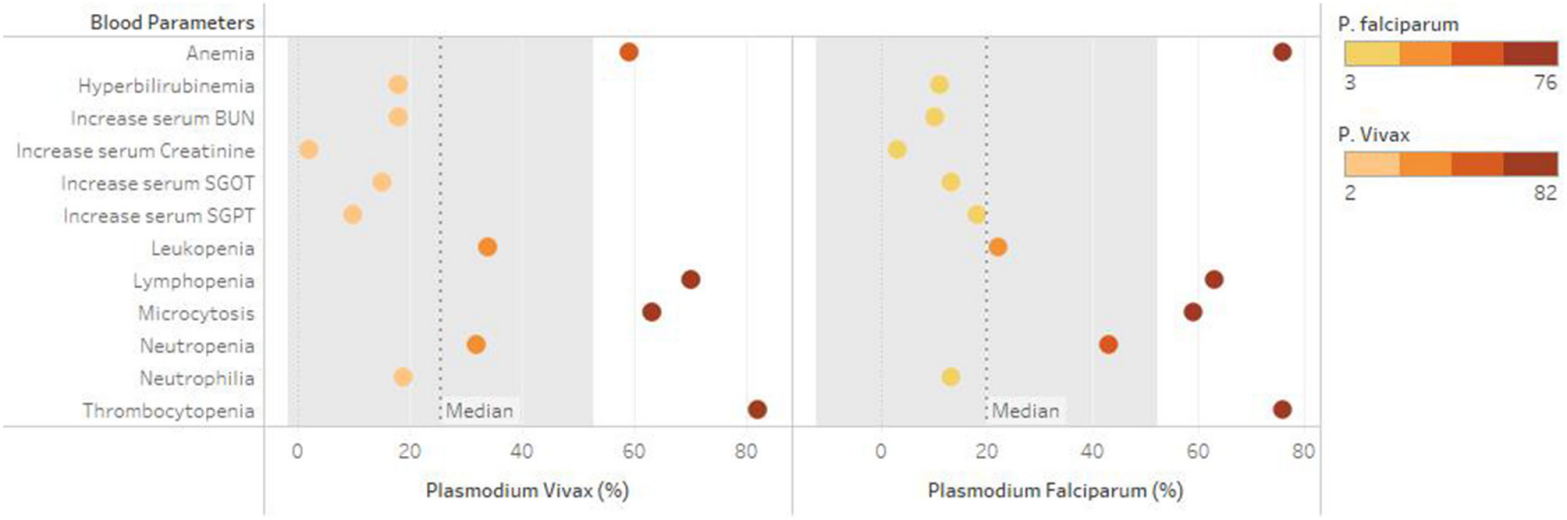
Commonly seen laboratory parameters among both *P. Vivax* and *P. falciparum*.

A total of 1,283 individuals were positive for malaria tested using different diagnostic techniques like microscopy, RDT, and both. The overall average malaria prevalence in the three groups was 8.2%. Among the enrolled patients, 96.8% (95.9–97.7% CI 95%) of the 1,283 patients tested positive for *P. vivax*, 2.1% (1.3–2.9 CI 95 %) for *P. falciparum*, and 14 (1.1%) with (0.5–1.6 CI 95 %) for mixed infection, as shown in Table 2. The average ratio of *P. vivax and P. falciparum* was 0.02. The prevalence of malaria in children under 5 years of age, pregnant women, and non-pregnant women were 23.3, 4.4, and 72.3%, respectively. Compared with other age groups, the prevalence was surprisingly high in non-pregnant women, as shown in Table 3. In terms of clinical manifestations, the most frequently observed symptoms in patients with *P. falciparum* were fever with chills and sweating. Nevertheless, the prominent symptoms of *P. vivax* infection were headache and fever with chills and myalgia, as shown in Figure 3. Different laboratory parameters were assessed in these patients; thrombocytopenia, anemia, and lymphopenia were most commonly observed, as shown in Figure 4. Anemia was most commonly seen in *P. falciparum*, while thrombocytopenia was reported in *P. vivax* patients. Other laboratory measures, including mean corpuscular volume, lymphocytes, neutrophils, urea, creatinine, SGPT, and bilirubin level, are more likely to be consistent in both species, as shown in Table 4.

**Table 2:** Prevalence of malaria infection in Bannu district (*n* = 15,650).

	**Positive**	**%**	**95 % CI**	
			**Lower**	**Upper**
*P. falciparum*	27	2.1	1.3	2.9
*P. vivax*	1,242	96.8	95.9	97.7
*P. falciparum + P. vivax*	14	1.1	0.5	1.6

**Table 3:** Prevalence of malaria infection by age and sex.

**Characteristics**	***P. falciparum***	***P. vivax***	***P. falciparum + P. vivax***	**Total**	***P. falciparum: P. vivax***
	***N*** **(%)**	***N*** **(%)**	***N*** **(%)**	***N*** **(%)**	
0–5 years age children	8 (2.68)	287 (95.99)	4 (1.34)	299 (23.30)	0.29
Pregnant women	1 (1.79)	55 (98.21)	0 (0)	56 (4.36)	0.05
Women	18 (1.94)	900 (96.98)	10 (1.08)	928 (72.33)	0.02
Total	27 (2.10)	1,242 (96.80)	14 (1.09)	1,283	0.02

**Table 4:** Blood parameters in *Plasmodium Vivax* and *Plasmodium Falciparum*.

**Blood parameters**	***P. falciparum***	***P. Vivax***
Hemoglobin (g/dL)	10.6 ± 2.3	12.3 ± 1.7
WBC (x 10^3^/μL)	8.2 ± 1.3	6.1 ± 3.5
Neutrophil (x 10^3^/μL)	4.2 ± 2.8	6.1 ± 3.5
MCV (fl)	77 ± 9.4	71 ± 11.3
Lymphocytes (x 10^3^/μL)	1.3 ± 0.9	1.29 ± 0.18
Platelets (x 10^3^/μL)	81.34 ± 33.2	97.4 ± 29.3
Bilirubin (mg/dL)	1.1 ± 1.4	1.4 ± 2.3
SGPT (IU/L)	53 ± 22.3	38 ± 19.8
BUN (mg/dL)	41 ± 11.2	47 ± 18.3
Creatinine (mg/dL)	0.7 ± 0.47	0.6 ± 0.32

The primary treatment used for malaria infection reported by the health centers was primaquine, followed by chloroquine (Table 5). The total treatment used for malaria infection in Bannu district was Artemisinin Combination Therapy (Artesunate + Sulfadoxine-Pyrimethamine) (ACT (AS+SP): 29 (0.1%), Chloroquine tablet: 16,896 (29.7%), Chloroquine Syrup: 44 (0.1%), Primaquine (PQ): 39,512 (69.4%), Artemisinin Combination Therapy (Artemether + Lumefantrine) ACT (ART+LF): 0.00%, and Tab Quinine: 0.8%.

**Table 5:** Malaria treatment in RDT health centers.

**Health centers**	**N**	**ACT (AS +SP)**	**Chloroquine**		**Primaquine**	**ACT (ART + LF)**	**Tab Quinine**	**Total**	**Chloroquine: Primaquine**
		***N*** **(%)**	**Tab *N* (%)**	**Syrup *N* (%)**	***N*** **(%)**	***N*** **(%)**	***N*** **(%)**		
Microscopy	18	2 (0.0)	5,421 (28.9)	2 (0.0)	13250 (70.6)	0 (0.0)	86 (0.5)	18,761	0.4
RDT	47	13 (0.1)	6,021 (29.2)	32 (0.2)	14,139 (68.7)	6 (0.0)	380 (1.8)	20,593	0.4
Microscopy + RDT	15	14 (0.1)	5,454 (31.0)	10 (0.1)	12,123 (68.9)	4 (0.0)	0 (0.0)	17,605	0.4
Total	80	29 (0.1)	16,896 (29.7)	44 (0.1)	39,512 (69.4)	10 (0.0)	466 (0.8)	56,959	0.4

*ACT (AS+SP), artemisinin combination therapy (artesunate + sulfadoxine – pyrimethamine); ACT (ART+LF), artemisinin combination therapy (artemether + lumefantrine); RDT, rapid diagnostic techniques; PCR, polymerase chain reaction*.

## Discussion

The incidence and prevalence of malaria can be effectively reduced through active and passive diagnosis (23). Accurate assessment of malaria infection may also help expand control interventions and malaria surveillance levels in Pakistan (24). In Bannu, Khyber Pakhtunkhwa, Pakistan, we executed a study to determine the malaria burden among pregnant women, children under the age of five, and healthcare facilities that provide malaria services to eradicate malaria in Pakistan.

Our study identified the burden of malaria for pregnant women, non-pregnant women, and children <5 years of age, and malaria service health facilities in Bannu district, Khyber Pakhtunkhwa, Pakistan. We found 27 (2.1%) with (1.3–2.9 CI 95%) positive for *P. falciparum*, 1,242 (96.8%) with (95.9–97.7 CI 95%) positive for *P. vivax*, and 14 (1.1%) with (0.5–1.6 CI 95%) showed mixed infection (*P. vivax* and *P. falciparum*). The average ratio of *P. vivax and P. falciparum* was 0.02. The prevalence of Malaria in Children under 5 years of age, pregnant women, and non-pregnant women were 23.3, 4.4, and 72.3%, respectively. High prevalence existed in non-pregnant women as compared to the other groups. The dominance of *P. vivax* is consistent with other parts carried out by Pakistan studies (13, 25–27), but our results differ from another study in East Balochistan (28). Another study also defined a high prevalence (10.8%) of *Plasmodium* infection and a high proportion of cases attributed to *P. falciparum* in Hangu, Thall, and Bannu districts of Khyber Pakhtunkhwa province (13). In other highly endemic districts of Khyber Pakhtunkhwa province (that were not part of our study), a comparatively high prevalence of *P. falciparum* was found, ranging from 16% in Buner (29) to 25% in Abbottabad and Bannu (30, 31). In 1979–82, Afghanistan refugees moved to Balochistan and the regions of Khyber Pakhtunkhwa across the border. In these places, cross-border migration could have helped to increase or sustain malaria infections (31–33).

Our study also shows a high prevalence of malaria in non-pregnant women, consistent with other studies in Pakistan (11), but differs from other studies (34, 35). The risk factors and reasons for the gender difference could not be explained because the study participants did not provide data on actual lifestyles. This type of information would be added as a study parameter in the future.

Malaria control requires the use of effective antimalarial drugs, including a comprehensive approach to prevention (mainly vector control) and early treatment. Since 2010, all *P. falciparum* endemic countries have gradually updated their treatment policies, from monotherapy using chloroquine, amodiaquine, and sulfadoxine-pyrimethamine to the currently recommended combination therapy based on artemisinin. Combination therapies are highly effective and well-tolerated, which reduced the mortality and morbidity of malaria worldwide. Unfortunately, *P. falciparum* in Southeast Asia has recently developed artemisinin resistance, threatening these gains (36).

According to our results, the primary treatment used for malaria infection reported by the health centers was primaquine, followed by chloroquine (Table 5). The total treatment used for malaria infection in Bnu Bannu district was Artemisinin Combination Therapy (Artesunate + Sulfadoxine-Pyrimethamine) (ACT (AS+SP): 29 (0.1%), Chloroquine tablet: 16,896 (29.7%), Chloroquine Syrup: 44 (0.1%), Primaquine (PQ): 39,512 (69.4%), Artemisinin Combination Therapy (Artemether + Lumefantrine) ACT (ART+LF): 0.00%, and Tab Quinine: 0.8%. Reports of 2004 suggest the cure rate of chloroquine treatment was 58% in Punjab and only 17% in Sindh and Balochistan provinces (37). The efficacy of sulfadoxine-pyrimethamine and amodiaquine in Balochistan was 44 and 47%, respectively. In 2004, the treatment rate of artesunate + sulfadoxine-pyrimethamine in the federally administered tribal areas was 97%. Similarly, the efficacy of artesunate + sulfadoxine-pyrimethamine tested in Balochistan, Federally Tribal Areas, and Sindh reached to 100% treatment rate in 2008. Similarly, in 2009, artemisinin and lumefantrine treatment rates in Khyber Pakhtunkhwa, Balochistan, and Sindh were also 100% (37).

In 1981, the resistance of antimalarial drugs to chloroquine was first discovered in the Sheikhupura district of Punjab. An analysis conducted by the National Institute of Malaria Research and Training from 1977 to 1995 showed R1 chloroquine resistance in Pakistan (the initial response to the drug was good, but 1 month after cure, parasitemia occurred within the frequency range of 30 to 84%. From 2004 to 2009, a plan evaluation of the efficacy of antimalarial drugs showed that resistance to chloroquine is common among falciparum malaria across the country, while resistance to sulfadoxine-pyrimethamine has reached its peak. In contrast, artemisinin-based combination therapy can 100% effectively treat uncomplicated falciparum malaria. Therefore this combination therapy is officially adopted as the first-line treatment for undiagnosed *falciparum* malaria (37).

Priority should be given to speeding efforts to improve the classification of asymptomatic malaria in high-risk areas and developing reliable diagnostic criteria for this kind of malaria infection. Continuous risk factor identification, upgraded epidemiological surveys, and better epidemiologic modelling must become integral components of all clinical investigations to aid in this approach. Additionally, significant effort should be made to develop extremely sensitive, affordable, and widely available rapid diagnostic detection kits that detect low parasitemia (12, 38).

Anemia is the most common distinguishing feature in both malarial species. Among the most frequent consequences of malaria infection is anemia, which affects children under the age of five and pregnant mothers living in high prevalent regions (39). The etiology of anaemia in malaria is unknown. However, the parasite's primary target is the red blood cell, may lead to the lysis of red blood cells upon releasing of malaria parasites, bone marrow dysfunction, and life span of circulating red cells (40, 41). Similar results has been reported by various studies, reported significant reduction of hemoglobin level among malarial patients (42–44). Malaria platelet abnormalities include both qualitative and quantitative changes. Platelet counts were found to be substantially lower in malaria patients in this investigation. Thrombocytopenia was observed in majority of malaria patients. These findings might suggest that thrombocytopenia is a sign of malaria, according to certain previous studies (45–47).

The research work has several limitations. The study was conducted in the endemic district (Bannu) of Khyber Pakhtunkhwa Province. Therefore, the results of the research work lack generalization to be considered for the whole country. In addition, due to the cross-sectional nature of the study, causality cannot be assumed in the association between risk factors and malaria prevalence. A more extended study should be conducted to determine the seasonal changes and other risk factors of the malaria epidemic in this region.

The findings of this study reveal that the prevalence of malaria in the non-pregnant women's group was more affected by malaria. *P. vivax* infection was found high than other malaria infections. The primary treatment used for malaria infection reported by the health centers was primaquine, followed by chloroquine. In the Bannu district, the health care facilities for malaria services appeared weak. There is a strong need for attention to those living in this district and reinforcing malaria control or elimination strategies.

## Data Availability Statement

The original contributions presented in the study are included in the article/supplementary files, further inquiries can be directed to the corresponding authors.

## Ethics Statement

The studies involving human participants were reviewed and approved the University of Science and Technology Bannu, Pakistan. Permission to conduct the research and relevant information was obtained by the relevant authorities at the Directorate of the Malaria Control Programme, Pakistan. Written informed consent to participate in this study was provided by the participants' legal guardian/next of kin.

## Author Contributions

HQ and MK were responsible for data collection and wrote the paper. AA, AK, and UA collected data. AK and YS review the paper. All authors contributed to the article and approved the submitted version.

## Funding

This work was supported by the Department of Imaging, Southern University of Science and Technology Hospital, Shenzhen, Guangdong, PR China.

## Conflict of Interest

The authors declare that the research was conducted in the absence of any commercial or financial relationships that could be construed as a potential conflict of interest.

## Publisher's Note

All claims expressed in this article are solely those of the authors and do not necessarily represent those of their affiliated organizations, or those of the publisher, the editors and the reviewers. Any product that may be evaluated in this article, or claim that may be made by its manufacturer, is not guaranteed or endorsed by the publisher.
